# A longitudinal quasi-experimental study of a pedagogical approach to supporting undergraduate well-being and mental health: digital interdisciplinary accredited elective mental health literacy university course

**DOI:** 10.1192/bjo.2025.10960

**Published:** 2026-02-16

**Authors:** Anne Duffy, Nathan King, Daniel Rivera, Kurtis Pankow, Simone Cunningham, Elizabeth Tetzlaff, Kristen Kyone, Emily Dephoure, Adeleine Lyon, Lucy Robinson, Edward Watkins, Charles Keown-Stoneman

**Affiliations:** Department of Psychiatry, Queen’s University, Kingston, Ontario, Canada; Department of Psychiatry, University of Oxford, Oxford, UK; Centre for Neuroscience Studies, Queen’s University, Kingston, Ontario, Canada; Department of Sport and Exercise, Swansea University, Swansea, UK; Department of Strategy, Entrepreneurship & International Business, Dalhousie University, Halifax, Nova Scotia, Canada; Life Sciences Program, Queen’s University, Kingston, Ontario, Canada; Department of Clinical Psychology, Newcastle University, Newcastle, UK; Department of Psychology, Exeter University, Exeter, UK; Dalla Lana School of Public Health, University of Toronto, Toronto, Ontario, Canada; Applied Health Research Centre (AHRC), Unity Health, Li Ka Shing Knowledge Institute, Toronto, Ontario, Canada

**Keywords:** Mental health literacy, anxiety, depression, well-being, university student mental health

## Abstract

**Background:**

Entry to higher education coincides with a period of accelerated psychosocial and brain development. Student need for acceptable and accessible well-being and mental health support is straining university resources.

**Aims:**

To evaluate the acceptability and impact of a digital mental health literacy course tailored for undergraduates and delivered as an accredited interdisciplinary elective.

**Method:**

Analyses included pre–post course survey data from enrolled students and longitudinal U-Flourish Well-Being Survey data from a comparison sample of non-course takers over the same period (2021–2024). Linear mixed-effects models examined associations between course participation and 12-week changes in mental health literacy, psychosocial risk factors, well-being and common mental health concerns.

**Results:**

Pre–post course survey data (*N* = 2884) supported high acceptability, improvements in resilience (+0.06; 95% CI 0.03–0.08, *p* < 0.001) and self-compassion (+0.65; 95% CI 0.46–0.84, *p* < 0.001), and a reduction in brooding (−0.31; 95% CI −0.44 to−0.18, *p* < 0.001). Taking the course was associated with a reduction in anxiety (*β* = −0.41; 95% CI −0.55 to −0.27, *p* < 0.001) and cannabis use (proportional odds ratio 0.82; 95% CI 0.75–0.90, *p* < 0.001), improvement in sleep quality (*β* = 0.79; 95% CI 0.61–0.97, *p* < 0.001) and evidence of a protective effect on well-being (*β* = 0.24; 95% CI 0.11–0.36, *p* < 0.001) and depressive symptoms (*β* = −0.37; 95% CI −0.52 to −0.21, *p* < 0.001), compared with non-course takers. Effects differed by gender, with women benefitting most, but were comparable across minoritised student subgroups.

**Conclusions:**

Mental health literacy delivered as an accredited undergraduate interdisciplinary course is highly acceptable and associated with improvement in psychological coping and positive effects on student mental health and well-being. Future research should focus on more diverse student samples, underlying mechanisms and sustained effects.

Student mental health is a focus of concern for higher education institutions and governments in both Canada and the UK.^1–3^ University provides the opportunity for young people at an important stage in their biological and psychosocial development to advance knowledge and scholarship, while learning more about themselves in a broader sociocultural context.^4^ Entry to university coincides with the peak period of risk for the onset of mental illness, which, if left untreated, can lead to chronic and more complex mental disorders, diminished academic performance, school drop-out, self-harm and suicide.^5^ Common mental disorders such as anxiety and depression have increased in young people over the past decade in keeping with trends in the general population.^6–8^ Despite only a small proportion of those students who could benefit reaching out for support, demand for mental health services continues to outpace enrolment and is putting significant strain on university resources.^4,9,10^ Effective, accessible, non-stigmatising and sustainable approaches to promote university student well-being and prevent common mental health concerns are essential to ensure a successful transition to and through university life, with enduring benefits for individuals and our global society.^5,11,12^

The transition to university is both an exciting and challenging time, as young people are tasked with leaving home, meeting higher education standards, navigating new social relationships and taking on more responsibility for managing their time, finances and lifestyle.^4^ Access to university has increased, with students from more diverse backgrounds attending, who may be more vulnerable to mental health problems in the transition to higher education.^13^ Recent evidence supports that socioemotional coping including psychological processes (i.e. self-compassion) and cognitive styles (i.e. negative rumination or brooding) and sleep quality influence mental health and academic outcomes in undergraduates.^14–17^ Further, stigmatising attitudes, low emotional self-awareness and a preference for self-reliance are student-reported barriers to proactive help-seeking, highlighting gaps that could be addressed through mental health literacy tailored to address these targets in undergraduate students.^9,10,13,18,19^

A non-stigmatising academic environment may be an effective setting to teach about mental health risk and protective factors, healthy socioemotional coping, early symptom recognition and appropriate help-seeking.^9^ Although there is some evidence to support the acceptability and short-term effectiveness of mental health literacy delivered in secondary schools,^20,21^ there is little reliable data to support substantive or sustained effects and a relative lack of reported evidence about potential harms for students.^22,23^ Studies of mental health literacy uptake and effectiveness in university students have been limited by highly variable interventions (delivery, focus and content), short follow-up periods and small sample sizes. Furthermore, very few studies have assessed potential mechanisms of action or the impact on student mental health.^23^ Our preliminary findings support that university undergraduate student-tailored mental health literacy offered as an accredited online (asynchronous) full semester course is a highly acceptable way of delivering this content and associated with improvement in sleep quality, emotional self-awareness and reducing barriers to help-seeking, including improving confidence and knowledge about where and when to reach out for support.^24^

## Aims

The primary aim of this longitudinal quasi-experimental pragmatic study was to evaluate the acceptability and effectiveness of a fully digitally integrated mental health literacy course offered as an accredited interdisciplinary elective to a large sample of undergraduate students. Objectives of the study were to (a) assess the acceptability of the mental health literacy course in a large sample of undergraduate students; (b) measure pre–post course changes in mental health literacy and psychosocial factors associated with mental health (i.e. stigmatising attitudes, emotional self-awareness, self-compassion, resilience, stress and brooding); (c) evaluate mental health and well-being outcomes associated with taking the course and (d) determine whether taking the course had differential effects for students from minoritised groups (i.e. based on ethnicity, gender, international student status, lifetime mental illness).

## Method

### Study design

This study included data from undergraduate students attending Queen’s University (Canada) who took the 12-week course ‘IDIS 199 The Science of Mental Health, Well-being, and Resiliency’ between Summer 2021 and Winter 2024 academic terms and consented to participate in this research. Details on the course development and content have been published elsewhere.^24^ Briefly, the mental health literacy course was designed as an accredited interdisciplinary 12-week online asynchronous undergraduate elective that incorporates state-of-the-art pedagogical and equity diversity inclusion principles and used a reverse mentorship approach in development. The course incorporates cognitive–behavioural therapy principles to facilitate the application of learning and support students to tackle problems and address barriers related to developing healthy lifestyle and coping, early recognition and self-management of anxiety and depressive symptoms, and appropriate and timely help-seeking.^24^

Students enrolled in the mental health literacy course were given the option to participate in research to evaluate the acceptability and effectiveness of the course. After reading a Letter of Information, consenting students were asked to complete an abbreviated electronic version of the U-Flourish Well-being Survey^25^ in the first week before starting the course (pre-course) and the last week of the 12-week term after completing the course (post-course). Students opting into research were incentivised by earning an additional 2% toward their course grade for completion of the pre–post course surveys. Students who did not wish to participate in research had the opportunity to earn an additional 2% course grade by completing a pre–post course self-reflection assignment that was not part of the research and required similar time and effort. The pre-course survey captured student demographic information and included validated measures of mental health literacy, psychosocial factors, well-being and common mental health symptoms. These measures were repeated in the post-course survey along with additional Likert-type and open-ended questions asking about course relevance, applicability and what was learned that was not known before. Optional (non-incentivised) follow-up surveys were offered to research participants at 8 and 12 weeks post-course, to assess sustained changes in mental health and well-being.

Students not enrolled in the mental health literacy course and who completed the biannual campus-wide U-Flourish Well-being Survey over the same term served as a non-randomised comparison sample. The U-Flourish Student Well-Being Survey content and procedures, including engagement activities and participation incentives, have been published elsewhere.^25^ The pre–post course surveys and the U-Flourish Well-Being Survey included the same set of validated measures. Both student course takers and non-course takers contributing data to this analysis provided informed consent after reading a Letter of Information outlining the study and recording their consent in the electronic survey. All procedures contributing to this work comply with the ethical standards of the relevant national and institutional committees on human experimentation and with the Helsinki Declaration of 1975, as revised in 2013. All procedures involving human patients were approved by the Queen’s Health Sciences Research Ethics Board (approval number HSREB-PSIY-739-22).

### Survey measures

#### Demographics

Age, gender (male, female, non-binary or prefer not to say) and international student status were self-reported at baseline. Students selected their ethnicity from a standard list, which for analysis were collapsed into: White, Asian, Black and Other (including multiple ethnicity). The highest level of education completed by either parent was used an indicator of socioeconomic status. Lifetime and family history of a mental illness diagnosis was reported at baseline.

#### Acceptability

Students were asked post-course to rate their level of agreement (1 = strongly disagree to 7 = strongly agree) with the following statements: (a) the course helped me be more aware of my well-being and mental health, (b) the course was engaging and effectively held my interest, (c) I will be able to apply what I learned to support my well-being and mental health, and (d) I would recommend this course to other students. Qualitatively, students were asked what three things they learned in the mental health literacy courses that were the most useful to their situation.

#### Mental health literacy

Students rated their level of agreement (from 1= strongly disagree to 5 = strongly agree) to each of nine items from the modified Mental Health Literacy Questionnaire (MHLQ) that mapped to two domains: knowledge of mental health problems (e.g. ‘Highly stressful situations my cause mental disorders’) and knowledge of self-help strategies (e.g. ‘Doing something enjoyable contributes to good mental health’).^26^ The measure of mental health knowledge was included on all iterations of the course survey and in the U-Flourish survey (Autumn 2021 to Winter 2022). Stigma barriers to care were assessed in course participants with the nine-item stigma subscale from the Barriers to Care Evaluation (BACE-3).^27^ Students were also asked to rank the following statements from the pre–post course surveys: ‘I am confident I know where to seek dependable information about my mental health and well-being’, and ‘I am confident I know how to access mental health support if needed (family doctor, counselling, online or over the phone advice)’ (1= strongly disagree to 5 = strongly agree).

#### Psychosocial factors associated with mental health

Emotional self-awareness was measured with the 11-item Emotional Self Awareness Scale (ESAS), assessing the ability to recognise and identify your own emotions.^28^ Self-compassion was measured with the six-item Self-Compassion Scale Short-Form (SCS-SF).^29^ Resilience was measured with the five-item Brief Resilience Scale.^30^ The five items were averaged to get a total score ranging from 1 to 5, with higher scores indicating greater resilience. Self-perceived stress over the past month was measured with the four-item Perceived Stress Scale (PSS-4).^31^ Brooding (i.e. negative rumination) was measured with the five-item Brooding subscale of the Rumination Response Scale.^32^ Lifetime suicidal thoughts and behaviours were assessed at baseline with items from the Columbia Suicide Rating Scale (CSRS).^33^

#### Mental health and well-being outcomes

Primary mental health outcomes were levels of anxiety, depression and well-being. Students reported symptoms of anxiety with the Generalised Anxiety Disorder-7 (GAD-7)^34^ and symptoms of depression with the Patient Health Questionnaire-9 (PHQ-9)^35^ over the past 2 weeks. On both scales, a score of 10 or more was used as a screening cut-off to identify clinically significant symptom levels. The eight-item Sleep Condition Indicator (SCI-8) measured sleep quality.^36^ Higher scores indicated greater sleep quality and a cut-off of 16 or less identified probable insomnia. Self-reported well-being over the past 2 weeks was measured with the seven-item Warwick–Edinburgh Mental Wellbeing Scale (WEMWBS-7),^37^ and a cut-off of 19 or less was used as an indicator of low well-being. Secondary outcomes were binge drinking frequency defined as five or more drinks on one occasion (rated as: never, less than monthly, monthly, weekly or daily to almost daily) and past month cannabis use frequency categorised into four groups: frequent (greater than 1–2 days per week), moderate (1–2 days per week), low (equal to or less than 1–3 days per month) or never.

### Statistical analysis

#### Acceptability and effectiveness in course takers – quantitative pre–post change

Pre–post course changes in mental health literacy and psychosocial factors was examined by using the absolute difference in scores, including the 95% confidence interval, and tested with a paired *t*-test. This analysis included students who completed both the baseline and follow-up surveys and had pre–post data on at least one measure of interest (*n* = 2399/2884; 83.2%). A total of 426 (14.6%) students were lost to follow-up and another 59 (2.0%) were missing pre–post data on all measures. This analysis was repeated stratifying by gender, although there was insufficient power to analyse the non-binary group. Person-mean imputation was used to calculate scale scores if a single item was missing. This analysis was conducted using 64-bit SAS version 9.4 (SAS Institute Inc., Cary, North Carolina, USA) on a Windows 11 platform; the software is available at https://www.sas.com/.

#### Effectiveness in course takers – qualitative feedback post-course survey

Qualitative data analysis followed a content analysis approach.^38^ A codebook was developed by a team of four; including one experienced qualitative researcher and three students. Following codebook development, the team divided the data into pairs and established interrater reliability on the first 50 open-text responses regarding what students had learned from the course. Interrater reliability was 98.9%. Following the resolution of coding discrepancies, the remaining data was divided and coded independently. A frequency count of codes was presented in a data matrix and data display.

#### Effectiveness in course takers compared with non-course takers

For the primary continuous outcomes, estimated effects were obtained from adjusted linear mixed-effects models, using repeated measures of the outcomes over the academic year and random intercepts for each subject. Secondary ordinal outcomes (i.e. binge drinking and cannabis use) were fit using proportional odds models, accounting for repeated measures with robust ‘sandwich’ standard errors, with effects reported as proportional odds ratios (PORs).^39^ Models were adjusted for time since baseline, semester and year, gender, international student, ethnicity, parental education, age, history of mental illness, family history of mental illness, lifetime suicide ideation, lifetime suicide attempt and lifetime self-harm. Interaction terms were included between time since baseline and course enrolment, to allow for different associations between the outcomes and time for course takers and non-course takers. For each subject, all outcome data available within 1 year (52 weeks) of the baseline measurement were included in the analyses. All longitudinal mixed-effects models and proportional odds models were fit with R version 4.3.2 for Windows 64 bit.^40^

For missing subject-specific covariate data in the adjusted models, 20 multivariate multiple imputed data-sets were generated by using multivariate imputation through chained equations with the *mice* package in R.^41,42^ Of the original 8004 students eligible for the analysis, 6859 had complete covariate data and 2884 had taken the course. After excluding those with no outcome data, 7647 students (i.e. 2880 course takers and 4847 non-course takers) contributed to the regression analyses. All individual covariates had less than 10.4% missingness. Based on the low percentages of missing covariate data, it was concluded that 20 imputed data sets were sufficient.^41^ To account for potential bias from who did or did not answer the outcome questions at each follow-up, inverse-probability weighting was used within each imputed data-set.^43,44^ The probabilities of completing each questionnaire based on the subject-specific covariate data were estimated using logistic regression models. Although time was measured to the precision of number of days since baseline, the effect estimates were obtained using predefined contrasts from the regression models for the estimated adjusted change in mean outcome values over a 12-week period from baseline (to represent the approximate length of a semester). Estimated adjusted mean differences at baseline from the adjusted mixed-effects models are also presented.

Further analyses were performed and presented to assess any differences in the effect of taking the course by specific demographic subgroups, with all two-way interactions between course taking, time since baseline and demographic group. Groups compared were divided on the basis of gender, ethnicity, age, international or domestic student, lifetime history of mental illness and term. Multivariate Wald tests were performed to assess the evidence that the adjusted effect of taking the course differed by each subgroup, by comparing the results from a model with all the two-way interactions between taking the course, time and subgroup, to a reduced model without the interaction terms with the subgroup. These models were adjusted for the same covariates as described above for the other mixed-effects models.

## Results

### Description of course takers

As summarised in Table 1, 2884 undergraduate students enrolled in the 12-week mental health literacy course between May 2021 and April 2024 and consented to participation in research (Supplementary Fig. 1 available at https://doi.org/10.1192/bjo.2025.10960). The majority of students taking the course were 21 years or younger, female, and from White or Asian ethnic backgrounds. Over 40% reported a lifetime history of a diagnosed mental illness. Over a third of students screened positive at baseline for clinically significant levels of anxiety (39%) and depressive symptoms (35%), and almost a fifth (19%) screened positive for low levels of well-being.

**Table 1:** Description of students who enrolled in the mental health literacy course and completed a course survey (*N* = 2884)

Baseline demographic characteristics and mental health	*n*	(%)
Age, years		
≤18	678	(23.7)
19–21	1494	(52.3)
≥22	684	(24.0)
Gender		
Male	630	(22.1)
Female	2184	(76.4)
Non-binary	29	(1.0)
Prefer not to say	14	(0.5)
Ethnicity		
White	1617	(57.0)
Asian	686	(24.2)
Black	93	(3.3)
Other (including multiple ethnicity)	441	(15.5)
International student		
Yes	105	(3.7)
No	2752	(96.3)
Parental education, highest level		
Graduate/postgraduate degree	993	(36.4)
Bachelor’s or trades/apprenticeship	1234	(45.3)
High school or less	498	(18.3)
Family history of mental illness		
Yes	1347	(51.6)
No	1264	(48.4)
Lifetime history of mental illness		
Yes	1201	(42.3)
No	1640	(57.7)
Anxiety screen positive (GAD-7 ≥10)	1106	(38.8)
Depression screen positive (PHQ-9 ≥10)	1005	(35.3)
Low well-being screen positive (WEMWBS-7 ≤19)	544	(19.1)
Insomnia screen positive (SCI-8 ≤16)	1000	(35.2)

GAD-7, Generalised Anxiety Disorder-7; PHQ-9, Patient Health Questionnaire-9; WEMWBS-7, Warwick–Edinburgh Mental Wellbeing Scale; SCI-8, Sleep Condition Indicator.

#### Acceptability of the mental health literacy course

As shown in Supplementary Table 1, students ranked the course (from 0 = strongly disagree to 7 = strongly agree) as being very helpful for developing awareness about their mental health and well-being (6.05 ± 1.20), engaging (5.79 ± 1.36) and applicable to supporting their mental health (6.06 ± 1.19). A large majority agreed that they would recommend the course to other students (6.26 ± 1.19). Less than 2% of students (0.9 to 1.7%) strongly disagreed with any of the acceptability questions.

Content analysis demonstrated that students endorsed learning about the importance of sleep for protecting and improving their well-being and mental health. The top five responses about new learning from the course beginning with most frequently reported were sleep, stress management (e.g. coping skills), study–life balance (e.g. making time for activities other than studying), exercise and mental health awareness (e.g. checking-in on how they were feeling) (Supplementary Table 2, Supplementary Fig. 2). Although most students limited their report to what they had learned, several students did report specific actions they had undertaken based on what they had learned, indicative of behavioural change (Supplementary Table 3). There was no evidence of direct harm related to taking the course from the student responses; however, a few students mentioned that the mid-term and final examinations of the mental health literacy course were scheduled close to examinations in other courses, which was stressful (e.g. scheduling issue).

#### Pre- to post-course changes in mental health literacy and psychosocial factors associated with well-being

The pre–post course changes in literacy and psychosocial factors associated with well-being and mental health in course takers who completed the baseline and follow-up surveys are summarised in Table 2. There was evidence of an increase in knowledge of mental health problems and self-help strategies, as well as an increase in knowledge of how and where to seek support after completing the course. There was also evidence of improvement after taking the mental health literacy course in student resilience and self-compassion, and a reduction in brooding. However, there was no evidence of a statistically significant change in stigmatising attitudes or in emotional self-awareness post course. Apart from resilience, which only improved in women, findings were consistent for both men and women (Supplementary Table 4).

**Table 2:** Pre- to post-course survey mental health literacy and psychosocial measures in course takers

Measures of mental health literacy and psychosocial factors	*n*	Pre-course (Time 1)		Post-course (Time 2)		Pre- to post-course change				
		Mean	(s.d.)	Mean	(s.d.)	Difference	(95% CI)	*t*-value	(d.f.)	*p*-value***
Mental health literacy										
Mental health knowledge (9–45)^a^	2297	39.5	(4.5)	40.4	(4.8)	0.92	(0.72–1.12)	8.87	(2296)	<0.001
Knowledge of mental health problems (5–25)^a^	2299	21.5	(3.0)	22.2	(3.0)	0.65	(0.52–0.78)	9.93	(2298)	<0.001
Knowledge of self-help strategies (4–20)^a^	2300	17.9	(2.1)	18.2	(2.3)	0.27	(0.18–0.36)	5.59	(2299)	<0.001
Know where to seek mental health Information (1 = strongly disagree to 5 = strongly agree)	2384	3.89	(0.9)	4.03	(0.8)	0.14	(0.11–0.18)	754	(2383)	<0.001
Know how to access mental health support (1 = strongly disagree to 5 = strongly agree)	2384	3.93	(0.9)	4.03	(0.8)	0.11	(0.07–0.14)	5.40	(2383)	<0.001
Stigma barriers to care (0–27)	2378	5.83	(6.3)	5.69	(6.4)	−0.14	(−0.36 to 0.09)	−1.18	(2377)	0.23
Psychosocial factors										
Emotional self-awareness (0–40)	2338	23.1	(5.1)	23.0	(4.9)	−0.12	(−0.28 to 0.05)	−1.36	(2337)	0.17
Self-compassion (6–30)	2377	18.6	(5.2)	19.3	(5.1)	0.65	(0.46–0.84)	6.76	(2376)	<0.001
Resilience (1–5)^a^	2293	3.13	(0.8)	3.19	(0.7)	0.06	(0.03–0.08)	4.32	(2292)	<0.001
Stress (0–16)	2386	7.29	(3.2)	7.61	(3.1)	0.32	(0.21–0.44)	5.66	(2385)	<0.001
Brooding (5–20)^b^	2031	11.9	(3.4)	11.6	(3.5)	−0.31	(−0.44 to 0.18)	−4.66	(2030)	<0.001

a. Not in summer 2021 term survey.

b. Not in summer 2021 or autumn 2021 surveys. ***p-value for paired *t*-test.

#### Associations between course participation and mental health literacy, psychosocial factors, and well-being and mental health outcomes (course takers versus non-course takers)

Comparing pre–post mental health literacy course data with data from the U-Flourish Well-Being Survey data from non-course takers over the same time period, linear mixed-effect models adjusted for covariates showed evidence that taking the course had a positive effect on understanding the determinants of mental health (0.95 units/12 weeks; 95% CI 0.71−1.19) and knowledge of how to seek help, when and if needed (0.31 units/12 weeks; 95% CI 0.19−0.42). Similarly, there was evidence that taking the course had a beneficial effect on reducing brooding (−0.35 units/12 weeks; 95% CI −0.46 to −0.23), improving self-compassion (0.41 units/12 weeks; 95% CI 0.27−0.55) and reducing stress (−0.12 units/12 weeks; 95% CI −0.21 to −0.04) (Table 3).

**Table 3:** Estimated adjusted association with taking the mental health literacy course on literacy, psychosocial factors and well-being and mental health (course takers compared with non-course takers)

Outcome	Baseline in course takers	Baseline in non-course takers	Difference at baseline	Slope in course takers (per 12 weeks)	Slope in non-course takers (per 12 weeks)	Estimated effect of the course (per 12 weeks)
Mental health literacy						
Mental health knowledge (9–45)	30.91 (30.65–31.17)	28.95 (28.61–29.29)	**1.96 (1.43–2.50) *p* < 0.001**	**0.93 (0.72–1.14)**	−0.02 (−0.14 to 0.10)	**0.95 (0.71–1.19) *p* < 0.001**
Knowledge of mental health problems (5–25)	16.67 (16.50–16.84)	15.36 (15.13–15.59)	**1.31 (0.96–1.66) *p* < 0.001**	**0.66 (0.52–0.79)**	0.01 (−0.07 to 0.09)	**0.65 (0.49–0.80) *p* < 0.001**
Knowledge of self-help strategies (4–20)	14.24 (14.12–14.36)	13.60 (13.44–13.75)	**0.65 (0.40–0.89) *p* < 0.001**	**0.28 (0.18–0.37)**	−0.03 (−0.09 to 0.03)	**0.31 (0.19–0.42) *p* < 0.001**
Psychosocial factors associated with well-being						
Emotional self-awareness (0–40)	22.86 (22.59–23.12)	21.98 (21.61–22.35)	**0.87 (0.30–1.44) *p* = 0.003**	0.08 (−0.03 to 0.19)	**0.19 (0.08–0.30)**	−0.11 (−0.27 to 0.05) *p* = 0.17
Self-compassion (6–30)	12.55 (12.33–12.77)	12.93 (12.75–13.11)	**−0.38 (−0.70 to −0.06) *p* = 0.02**	**0.46 (0.35–0.57)**	0.05 (−0.04 to 0.14)	**0.41 (0.27–0.55) *p* < 0.001**
Resilience (1–5)	3.12 (3.08–3.15)	3.09 (3.07–3.12)	0.02 (−0.02 to 0.07) *p* = 0.34	**0.04 (0.03–0.06)**	**0.03 (0.02–0.04)**	0.01 (−0.01 to 0.03) *p* = 0.19
Stress (0–16)	7.21 (6.89–7.56)	7.24 (6.88–7.61)	−0.04 (−0.23 to 0.16) *p* = 0.73	0.03 (−0.04 to 0.09)	**0.15 (0.09–0.21)**	**−0.12 (−0.21 to −0.04) *p* = 0.006**
Brooding (5–20)	7.02 (6.86–7.17)	6.55 (6.39–6.72)	**0.47 (0.21–0.72) *p* < 0.001**	**−0.39 (−0.47 to −0.30)**	−0.04 (−0.12 to 0.04)	**−0.35 (−0.46 to −0.23) *p* < 0.001**
Mental health and well-being						
Anxiety symptoms (GAD-7; 0–21)	8.42 (8.20–8.65)	8.55 (8.37–8.73)	−0.13 (−0.45 to 0.20) *p* = 0.44	**−0.14 (−0.25 to −0.03)**	**0.26 (0.18–0.35)**	**−0.41 (−0.55 to −0.27) *p* < 0.001**
Depressive symptoms (PHQ-9; 0–27)	8.27 (8.03–8.52)	8.33 (8.14–8.53)	−0.06 (−0.41 to 0.29) *p* = 0.74	0.04 (−0.08 to 0.16)	**0.40 (0.31–0.50)**	**−0.37 (−0.52 to −0.21) *p* < 0.001**
Sleep quality (SCI-8; 0–32)	19.78 (19.47–20.10)	20.49 (20.23–20.75)	**−0.70 (−1.17 to −0.24) *p* = 0.003**	**0.28 (0.14–0.42)**	**−0.50 (−0.62 to −0.39)**	**0.79 (0.61–0.97) *p* < 0.001**
Well-being (WEMWBS-7; 7–35)	23.48 (23.28–23.69)	22.98 (22.82–23.14)	**0.50 (0.22–0.79) *p* < 0.001**	0.06 (−0.04 to 0.15)	**−0.18 (−0.26 to −0.11)**	**0.24 (0.11–0.36) *p* < 0.001**

GAD-7, Generalised Anxiety Disorder-7; PHQ-9, Patient Health Questionnaire-9; SCI-8, Sleep Condition Indicator; WEMWBS-7, Warwick–Edinburgh Mental Wellbeing Scale.

Effects estimated from linear mixed-effects models adjusting for time since baseline, semester and year, gender, international student status, ethnicity, parental education, age, history of mental illness, family history of mental illness, lifetime suicide ideation, lifetime suicide attempt, and lifetime self-harm, with 95% CIs in parentheses. Statistically significant differences in mean baseline values, slopes and differences in slopes are bolded (*p* < 0.05). Sample sizes by outcome ranged from 2779 to 2880 for course takers, except for brooding (*n* = 2577). Sample sizes by outcome for non-course takers ranged from 4369 to 4788, except for the mental health literacy outcomes (2357–2358), emotional self-awareness (2489) and brooding (2777), which were not measured on all surveys.

**Fig. 1:**
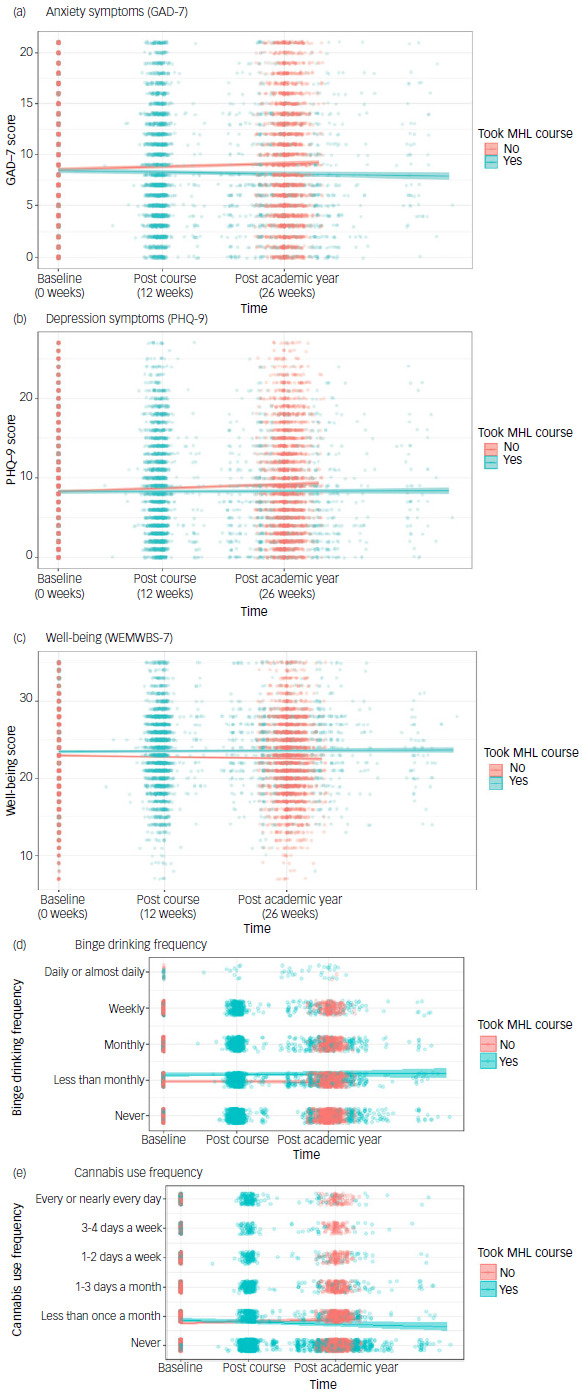
Plot of (a) estimated mean anxiety symptom GAD-7 scores, (b) mean depression symptom PHQ-9 scores, (c) mean well-being WEMWBS-7 scores, (d) mean binge drinking response and (e) mean cannabis use frequency, from adjusted linear mixed-effects models (solid lines in (a)–(c)) and adjusted proportional odds models (solid lines in (d)–(e)) in course takers (*n* = 2880) and non-course takers (*n* = 4840). Raw data are presented as translucent dots with a small amount of jitter to make overlapping values more visible. GAD-7, Generalised Anxiety Disorder-7; MHL, mental health literacy; PHQ-9, Patient Health Questionnaire-9; WEMWBS-7, Warwick–Edinburgh Mental Wellbeing Scale.

As shown in Table 3, after adjustments, mean anxiety symptom (GAD-7) scores lowered in students who took the mental health literacy course, but increased in students who did not take the course (Fig. 1, Table 3) over the same period. Adjusted mean anxiety symptom scores at baseline were comparable between course and non-course takers, with a mean difference of −0.13 units (95% CI −0.45 to 0.20; *p* = 0.44). There was no statistically significant benefit associated with the course on student reported resilience or emotional self-awareness after adjustments.

After adjustments, taking the mental health literacy course was associated with a reduction in anxiety symptoms (−0.14 units/12 weeks; 95% CI −0.25 to −0.03), whereas anxiety in non-course takers increased over the same period (Fig. 1, Table 3). Overall, taking the course was associated with a beneficial reduction in anxiety symptoms (−0.41 units/12 weeks; 95% CI −0.55 to −0.27; *p* < 0.001). Taking the mental health literacy course was associated with a protective effect against rises in depressive symptoms seen in students who did not take the course (−0.37 units/12 weeks; 95% CI −0.52 to −0.21; *p* < 0.001). Specifically, despite comparable baseline levels, non-course takers reported an increase in depressive symptoms (+0.40 units/12 weeks; 95% CI 0.31−0.50) not observed in course takers (0.04 units/12 weeks; 95% CI −0.08 to 0.16) (Fig. 1, Table 3). Sleep quality at baseline was lower in course takers compared with non-course takers, and improved in students taking the course, but worsened in non-course takers (Table 3). Overall, taking the mental health literacy course was associated with a 0.79 unit improvement in sleep quality score compared with non-course takers over 12 weeks (95% CI 0.61−0.97).

There was evidence that taking the course was associated with a protective effect on well-being (+0.24 units/12 weeks; 95% CI 0.11−0.36). That is, although there was no significant change in adjusted average well-being scores over the semester in students who took the mental health literacy course, there was a significant decline in well-being reported by non-course takers (Fig. 1, Table 3). Students who took the mental health literacy course had higher adjusted mean well-being scores at baseline compared with that of non-course takers (0.50 units; 95% CI 0.22−0.79; *p* < 0.001).

After adjustments, there was evidence that course takers were more likely to binge drink at baseline than non-course takers (POR = 1.41; 95% CI 1.20−1.65; *p* < 0.001), but there was insufficient evidence that taking the course was associated with changes in binge drinking frequency (POR = 1.03; 95% CI 0.95−1.12; *p* = 0.430). There was evidence that course takers were using cannabis more frequently than non-course takers at baseline (POR = 1.19; 95% CI 1.00−1.42; *p* = 0.046), and that taking the course had a beneficial effect on reducing cannabis use (POR = 0.82; 95% CI 0.75−0.90; *p* < 0.001). Frequency of cannabis use significantly decreased in course takers (POR = 0.90; 95% CI 0.83−0.98), whereas it significantly increased in non-course takers (POR = 1.09; 95% CI 1.05−1.14). Results from the substance use questions, including the estimated baseline probability for each ordinal level, are shown in Table 4.

**Table 4:** Estimated adjusted association with taking the mental health literacy course on binge drinking and cannabis use (course takers compared with non-course takers)

Outcome	Levels	Baseline probabilities in course takers	Baseline probabilities in non-course takers	POR at baseline	POR in course takers (per 12 weeks)	POR in non-course takers (per 12 weeks)	Estimated POR of course (per 12 weeks)
Binge drinking frequency	Never	0.33 (0.30–0.35)	0.41 (0.39–0.43)	**1.408 (1.203–1.648)** ***p* < 0.001**	1.023 (0.951–1.102)	0.990 (0.956–1.026)	1.033 (0.952–1.121) *p* = 0.430
	Less than monthly	0.34 (0.32–0.35)	0.33 (0.32–0.34)				
	Monthly	0.21 (0.19–0.22)	0.17 (0.16–0.18)				
	Weekly	0.12 (0.10–0.13)	0.09 (0.08–0.09)				
	Daily or almost daily	0.01 (0.01–0.01)	0.01 (0.00–0.01)				
Cannabis use frequency	Never	0.60 (0.57–0.63)	0.64 (0.62–0.66)	**1.192 (1.003–1.416)** ***p* = 0.046**	**0.897 (0.825–0.975)**	**1.094 (1.054–1.135)**	**0.820 (0.748–0.898)** ***p* < 0.001**
	Less than once a month	0.16 (0.15–0.17)	0.15 (0.14–0.16)				
	1–3 days a month	0.11 (0.10–0.12)	0.10 (0.09–0.11)				
	1–2 days a week	0.05 (0.05–0.06)	0.05 (0.04–0.05)				
	3–4 days a week	0.03 (0.03–0.04)	0.03 (0.02–0.03)				
	Every or nearly every day	0.04 (0.03–0.05)	0.04 (0.03–0.04)				

POR, proportional odds ratio.

Effects estimated from proportional odds models, accounting for repeated measures with robust standard errors, adjusting for time since baseline, semester and year, gender, international student status, ethnicity, parental education, age, history of mental illness, family history of mental illness, lifetime suicide ideation, lifetime suicide attempt and lifetime self-harm, with 95% CIs in parentheses. Statistically significant differences in mean baseline values, slopes and differences in slopes are bolded (*p* < 0.05).

#### Effectiveness of the mental health literacy course on mental health and well-being outcomes by diversity of student demographics and history

From multivariate Wald tests (Supplementary Table 5a), there was evidence that the effect of taking the mental health literacy course on anxiety differed by gender (*p* < 0.001), with a statistically significant decrease in adjusted mean GAD-7 scores observed only in women (Supplementary Table 6) Similarly, the effect of taking the course on reported levels of depressive symptoms (*p* < 0.001) and well-being (*p* = 0.009) also differed by gender. That is, statistically significant decreases in adjusted mean PHQ-9 scores were observed in female students and students who did not disclose gender, but not in male or non-binary students. Further, a statistically significant increase in adjusted mean well-being scores was only reported in women, without evidence of an effect in men, those who did not disclose gender and non-binary students. A positive effect of taking the mental health literacy course on sleep quality was observed across all genders, but differed in magnitude and did not reach statistical significance in non-binary students (Supplementary Table 6). Although not formally tested, it should be noted that at baseline in both course-taking and non-course-taking groups, female students appeared to have higher levels of anxiety (GAD-7) and depression (PHQ-9) compared with male students (Supplementary Table 5b).

There was evidence that the effect of the course on well-being differed by age (*p* = 0.038), although positive effects were observed in both younger and older age students (Supplementary Table 6). There was also evidence that the effect of the course on anxiety symptoms differed by semester (*p* = 0.01; Supplementary Table 6); specifically, the effect appeared to be stronger over time, with the largest decreasing effect in the Autumn 2023 semester (compared with Autumn 2021 and Autumn 2022). There was also evidence that the effect of the course on symptoms of depression differed by term (*p* = 0.04), although none of the effects for a specific term reached statistical significance (Supplementary Table 6).

There was insufficient evidence that the effect of the mental health literacy course on symptoms of anxiety and depression, sleep quality or well-being differed by ethnicity, international student status or lifetime mental illness (all *p* > 0.05) (Supplementary Table 5a).

## Discussion

### Principal findings

This longitudinal prospective study investigated the acceptability and impact of an accredited digitally integrated mental health literacy course for undergraduate university students. The course was designed and offered as a 12-week (one-term) asynchronous accredited elective suitable for students from different learning programmes. The course was very popular with undergraduate students as evidenced by full enrolment each semester that it was offered and low drop-out rates. Further, the course was ranked as highly acceptable, engaging and helpful by most students, with no evidence of reported harm. Although mental health literacy was quite high at baseline, students taking the course reported improvement in their knowledge of mental health problems and self-help strategies after having completed the course. Post-course, students reported improved levels of healthy psychological coping, including resilience and self-compassion, and lower levels of brooding and stress. Evidence supported that taking the course had a positive impact on student well-being and mental health outcomes. Specifically, students who took the course showed improved sleep, reduced anxiety and cannabis use, and with evidence of protection against the increase in depressive symptoms and reduction in well-being observed in non-course takers over the same 12-week period. Women benefitted more from the course in terms of reported mental health and well-being outcomes compared with men; students identifying as non-binary did not show the same benefits. There was no evidence of differences in acceptability or effectiveness of the course across minoritised groups identified on the basis of ethnicity, international student status, or having a lifetime mental illness.

### Alignment with literature

Our findings align with published research and provide evidence that improved knowledge about the determinants of mental health and well-being, healthy coping, having a better understanding of early symptoms and how to proactively seek help when needed promotes university student well-being.^45,46^ Current evidence suggests school-based mental health literacy improves knowledge, with small positive effects on stigma reduction and improved emotional self-awareness.^23,47^ Further, others have reasoned that providing mental health literacy in the transition to higher education can better prepare students, reduce stress and improve help-seeking at a critical time in psychosocial and brain development.^48^ Findings from our study support this hypothesis, showing evidence of improvement in key psychological factors and sleep quality^16,49^ known to be associated with anxiety and depression in university students; specifically, providing evidence of reduced brooding,^17^ and improved resilience and self-compassion.^50^ In this study, women benefitted more from taking the course than men, the former of whom had higher baseline levels of mental health symptoms. This finding therefore could represent a ceiling effect on improvement in men or the course was less effective and engaging for men. This finding underscores the need to investigate further how the course could address imbalances in gender outcomes.

Furthermore, there is supportive evidence that digital mental health literacy interventions are acceptable, effective, scalable and sustainable ways to engage students in learning about mental health; thereby empowering students, reducing access barriers and building support capacity.^51^ Research providing reliable to scale estimates of the impact of universal mental health literacy programmes on student mental health and well-being is still preliminary, and there is little understanding of the underlying mechanisms.^52^ Prior studies have been limited by small highly selected samples of students from specific learning backgrounds, a focus on short-term outcomes limited to knowledge, stigma and help-seeking. Mental health outcomes associated with mental health literacy has largely been limited to positive psychology and mindfulness.^23,46,53,54^

### Strengths and limitations of the study

This study included a large sample of undergraduate students from different learning backgrounds enrolled in the course; the majority of whom opted into research completing pre–post course research surveys with a low rate of missingness. Psychosocial factors and mental health and well-being outcomes were assessed using validated measures and repeated before and after having completed the course. Having a large comparison sample of undergraduate students (non-course takers) from the same institution who completed the same measures over the same time period, allowed us to estimate the effect of course participation. Important covariates affecting mental health literacy and mental health and well-being outcomes were assessed prospectively in the analysis. However, despite the large sample and high completion rates the sample included disproportionately more female students, mostly from White or Asian ethnic backgrounds, with male and international students under-represented compared with the university’s undergraduate census data. Further, there was limited power to assess acceptability and effectiveness of the course in students identifying as non-binary and prefer not to say gender, which contributed to wide confidence intervals and inconclusive null findings in some outcomes. This study relied on self-report measures of mental health and well-being with no direct observation or clinical assessment. Finally, although the comparison group completed the same validated measures over the same term, students were not randomised to conditions (i.e. to course or non-course takers), thereby limiting the ability to make a strong causal inference as to the effects of taking the course.

### Implications of findings

Findings provide robust support for the acceptability, feasibility and relevance of offering mental health literacy as an accredited interdisciplinary course tailored to undergraduate students. The results suggest that mental health literacy may be a scalable and effective way to promote healthy socioemotional coping and lifestyle change at scale; specifically, improving resilience, self-compassion, brooding and sleep quality. Further, evidence from this study supports that the mental health literacy course reduced anxiety and improved sleep quality, while protecting against increases in depressive symptoms and reduced well-being. Therefore, investing in university student-tailored mental health literacy offered as an accredited elective undergraduate course has potential as an acceptable, effective, scalable and sustainable proactive educational intervention.

### Future directions

A key next step would be to carry-out a controlled trial in which comparison is made between students randomised to the mental health literacy course or to a wait-list control or to an alternative course that omits key elements (e.g. the cognitive–behavioural components) to provide proof-of-principle that the mental health literacy course has a causal effect on mental health and well-being outcomes. Further there is a need to evaluate mechanisms underlying the positive effects of mental health literacy as a non-stigmatising educational intervention and to estimate both the short and long-term impacts on university student well-being and mental health. Further exploration and iterative content tailoring to meet the preferences and needs of more diverse higher education students is also needed. Finally, different versions of shorter duration could be developed and evaluated for specific target groups (i.e. faculty and staff) and purposes (i.e. university preparation course, high-risk student subgroups).

In conclusion, evidence from this longitudinal pragmatic study is consistent with the hypothesis that mental health literacy tailored for undergraduate students and offered as an accredited elective (i.e. credit bearing) is an acceptable and effective universal preventive intervention. Taking the course enhances student resilience and psychological coping, improves anxiety and sleep quality, and protects against worsening of depressive symptoms and well-being.

## Supporting information

Duffy et al. supplementary material Duffy et al. supplementary material

## Data Availability

The de-identified data that support the findings of this study are available from the corresponding author (A.D.) upon request.

## Supplementary material

The supplementary material is available online at https://doi.org/10.1192/bjo.2025.10960
