# Supplementary material for: A longitudinal quasi-experimental study of a pedagogical approach to supporting undergraduate well-being and mental health: digital interdisciplinary accredited elective mental health literacy university course
Source: BJPsych Open. 2026 Feb 16;12(2):e64. doi: 10.1192/bjo.2025.10960 (PMC12926893; doi:10.1192/bjo.2025.10960)
Supplement: Duffy et al. supplementary material [file S2056472425109605sup001.docx]

**Supplementary Figure 1**. Flow of the number of students who enrolled in the mental health literacy course and participated in the research component

Initially Enrolled:

**n= 2,908**; 98% of enrolment limit

Added course before add deadline: **n= 198**

Dropped course before drop deadline: **n= 107**

Enrolled at course add/drop deadline:

**n= 2,999**; 101% of enrollment limit

Consented & Completed a Survey:

**n= 2,884 (96.2%)**

Participated in Baseline & Follow-up Surveys: **n= 2,407/2,884 (83.5%)**

*By Term (n):*

Summer 2021: 85/96 (88.5%)

Fall 2021: 259/302 (85.8%)

Winter 2022: 205/232 (88.4%)

Summer 2022: 139/197 (70.6%)

Fall 2022: 303/355 (85.4%)

Winter 2023: 330/370 (89.2%)

Summer 2023: 240/309 (77.7%)

Fall 2023: 431/518 (83.2%)

Winter 2024: 439/505 (86.9%)

| **Supplementary Table 1**. Student course feedback rankings (*1= Strongly Disagree to 7= Strongly Agree*) | | | | | |
| --- | --- | --- | --- | --- | --- |
|  | **n** | **M** | **(SD)** | **Mdn** | **IQR** |
| The course helped me be more aware of my well-being and mental health | 2391 | 6.05 | (1.20) | 6 | (5-7) |
| The course was engaging and effectively held my interest | 2391 | 5.79 | (1.36) | 6 | (5-7) |
| I will be able to apply what I learned from this course to my well-being and mental health | 2389 | 6.06 | (1.19) | 6 | (5-7) |
| I would recommend this course to other students | 2392 | 6.29 | (1.19) | 7 | (6-7) |
| *Notes: M=Mean, SD= Standard Deviation, Mdn= Median, IQR= Interquartile Range* | | | | | |

| **Supplementary Table 2**. Frequency of thematic codes identified via content analysis (*3 things learned that were most useful to your situation*) | |
| --- | --- |
| **Code** | **# reporting** |
| Sleep | 764 |
| Stress management | 271 |
| Balance | 252 |
| Exercise | 233 |
| Mental health awareness | 216 |
| Social connections | 174 |
| Alcohol use | 142 |
| Substance use | 133 |
| Mental health services | 132 |
| Nature | 95 |
| Help-seeking | 89 |
| Nutrition | 86 |
| Self-care | 80 |
| Recreation | 80 |
| Self-regulation | 74 |
| Social prescribing | 57 |
| Resilience | 55 |
| Mindfulness | 54 |
| Cannabis use | 54 |
| Routine | 45 |
| Well-being | 43 |
| Stigma | 41 |
| Self-awareness | 39 |
| Academics | 27 |
| Self-compassion | 27 |
| Physical health awareness | 25 |
| Caffeine use | 23 |
| Culture | 21 |

**Supplementary Figure 2.** Word cloud summarizing the most useful new learning from the course reported by students. *Words are to scale based on the frequency of the codes.*


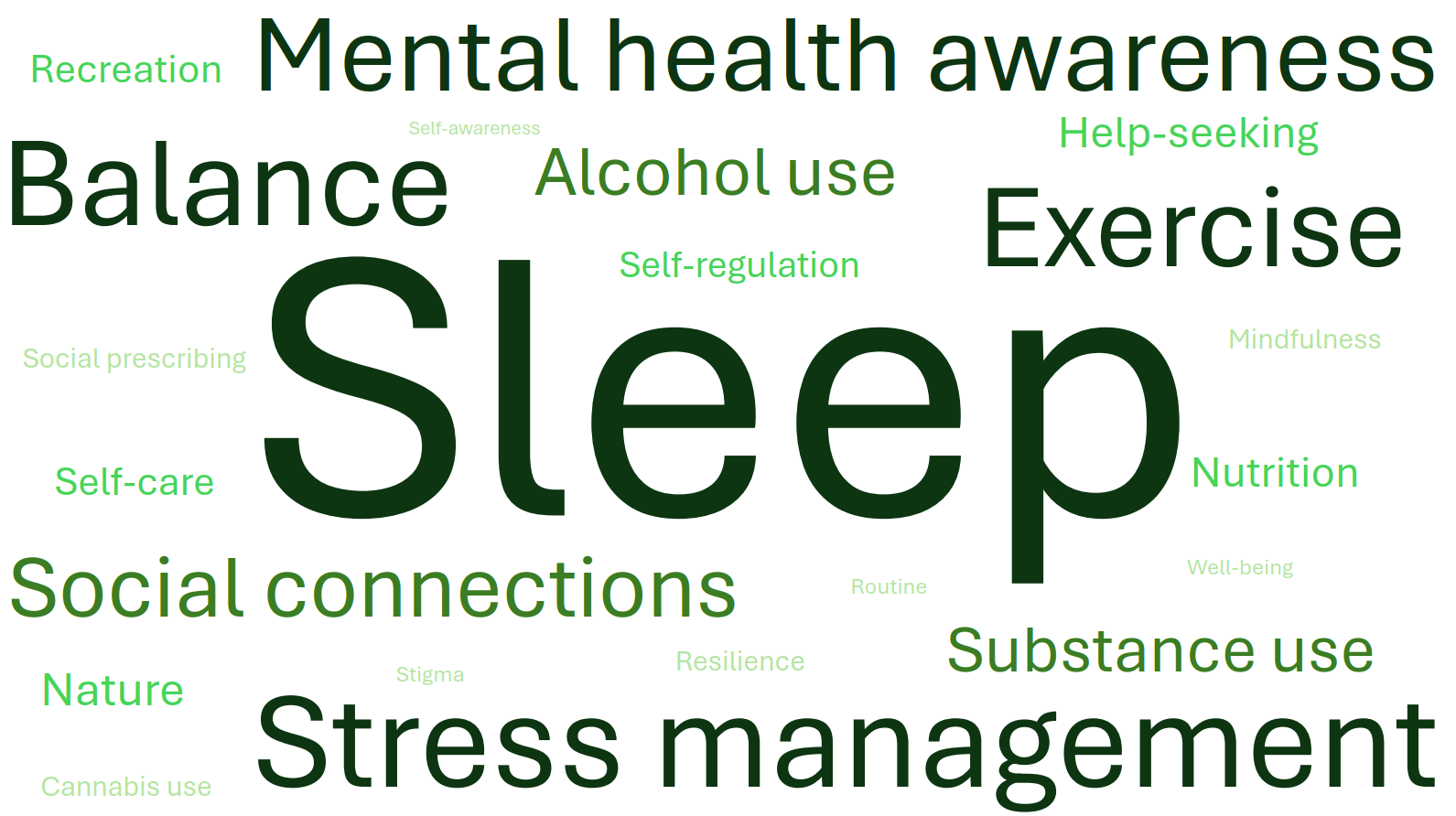


| **Supplementary Table 3.** Quotes illustrating evidence of behaviour change by course takers (*3 things you learned that were most useful to your situation*) | |
| --- | --- |
| Sleep-related behaviour changes | “I have taken up meditation before bed, I have tried to start implementing a better bedtime routine and making my sleeping environment more relaxing.” |
| Social connections-related behaviour changes | “During busy times in my life I would never prioritize relationships because I did not think they bettered my mental health. In fact, in the past I have decreased friendship time because I thought it was a distraction. Learning about how important friendships are made me prioritize them again, and I am so much happier.” |
| Exercise-related behaviour changes | “The value of exercise on mental and physical health: I got a gym membership, I feel happier, more alert, more physically healthy, and better about myself overall.” |
| Alcohol use-related behaviour changes | “I also learned about the dangers of alcohol use which has made me cut back on drinking.” |
| Help-seeking-related behaviour changes | “I have also learned about the importance of getting help and support. I usually tend to ignore my mental health problems because that’s what I saw growing up. I am now … getting the support I need.” |

| **Supplementary Table 4**. Pre- to post-course changes in psychosocial factors and mental health literacy in course takers, by gender | | | | | | | | | | | | | |
| --- | --- | --- | --- | --- | --- | --- | --- | --- | --- | --- | --- | --- | --- |
|  | | **Males** | | | | | | **Females** | | | | | |
|  | | **n** | **Pre-Course** | | **Post-Course** | | **p***** | **n** | **Pre-Course** | | **Post-Course** | | **p***** |
| **Psychosocial Factors** | |  | **Ⴟ** | **(SD)** | **Ⴟ** | **(SD)** |  |  | **Ⴟ** | **(SD)** | **Ⴟ** | **(SD)** |  |
| Resilience (1 to 5)* | | 515 | 3.40 | (0.80) | 3.40 | (0.78) | .99 | 1748 | 3.06 | (0.79) | 3.13 | (0.78) | <.001 |
| Self-Compassion (6 to 30) | | 522 | 19.1 | (5.3) | 19.8 | (5.1) | .001 | 1823 | 18.5 | (5.1) | 19.1 | (5.1) | <.001 |
| Emotional Self-Awareness (0 to 40) | | 510 | 22.9 | (4.9) | 22.6 | (4.9) | .23 | 1797 | 23.2 | (5.1) | 23.1 | (4.9) | .43 |
| Brooding (5 to 20)** | | 475 | 10.96 | (3.23) | 10.77 | (3.36) | .17 | 1527 | 12.25 | (3.41) | 11.88 | (3.52) | <.001 |
| Stress (0-16) | | 523 | 6.59 | (3.15) | 6.88 | (2.97) | .03 | 1831 | 7.47 | (3.19) | 7.79 | (3.13) | <.001 |
| **Mental Health Literacy** | |  |  |  |  |  |  |  |  |  |  |  |  |
| Mental Health Knowledge (9 to 45)* | | 516 | 39.2 | (4.7) | 40.0 | (5.0) | .002 | 1752 | 39.6 | (4.4) | 40.6 | (4.6) | <.001 |
|  | Mental Health Problems (5 to 25)* | 517 | 21.4 | (3.0) | 22.0 | (3.1) | <.001 | 1753 | 21.6 | (3.0) | 22.3 | (3.0) | <.001 |
|  | Self-Help Strategies (4 to 20)* | 517 | 17.9 | (2.3) | 18.0 | (2.4) | .19 | 1754 | 18.0 | (2.1) | 18.3 | (2.2) | <.001 |
| Know where to seek mental health Information (*1= strongly disagree to 5= strongly agree*) | | 522 | 3.90 | (0.87) | 4.00 | (0.81) | .01 | 1831 | 3.88 | (0.91) | 4.04 | (0.82) | <.001 |
| Know how to access mental health support (*1= strongly disagree to 5= strongly agree*) | | 522 | 3.93 | (0.93) | 3.98 | (0.86) | .25 | 1831 | 3.92 | (0.96) | 4.05 | (0.86) | <.001 |
| Stigma barriers to care (0 to 27) | | 520 | 5.60 | (6.09) | 5.56 | (6.19) | .87 | 1827 | 5.84 | (6.31) | 5.68 | (6.43) | 0.21 |
| **not in summer 2021 term survey, **not in summer 2021 or fall 2021 surveys, ***p-value for paired t-test, Ⴟ= Mean, SD= Standard Deviation* | | | | | | | | | | | | | |

| **Supplementary Table 5a.** Tests for differing mental health outcome course associations by diverse student subgroups | | | | | | |
| --- | --- | --- | --- | --- | --- | --- |
| **Outcome:** | **Gender** | **Ethnicity** | **Age** | **International vs Domestic Student Status** | **Lifetime History of Diagnosed Mental Illness** | **Semester** |
| Anxiety Symptoms (GAD-7) | **0.001** | 0.699 | 0.060 | 0.129 | 0.736 | **0.008** |
| Depressive Symptoms (PHQ-9) | **<0.001** | 0.291 | 0.074 | 0.401 | 0.293 | **0.041** |
| Well-being (SWEMWBS) | **0.009** | 0.285 | **0.038** | 0.987 | 0.850 | 0.138 |
| Sleep Quality (SCI-8) | **0.001** | 0.776 | 0.066 | 0.150 | 0.353 | **0.004** |

*P-values for multivariate Wald tests performed to assess whether the adjusted effect of taking the course differed by subgroup, by comparing the pooled results from a model with all the two-way interactions between taking the course, time, and the subgroup, to a reduced model without the interaction terms with the subgroup*

| **Supplementary Table 5b.** Adjusted baseline (pre-course) mental health outcome estimates by course enrolment and gender, with 95% confidence intervals | | | |
| --- | --- | --- | --- |
| **Outcome:** | **Gender:** | **Course** | **Non-Course** |
| Anxiety Symptoms (GAD-7) | Male | 7.08 (6.67, 7.50) | 6.91 (6.56, 7.26) |
|  | Female | 8.80 (8.55, 9.04) | 8.99 (8.78, 9.20) |
|  | Non-binary | 8.43 (6.42, 10.43) | 9.23 (8.20, 10.27) |
|  | Prefer not to say | 7.71 (4.95, 10.47) | 8.03 (6.16, 9.90) |
| Depressive Symptoms (PHQ-9) | Male | 7.31 (6.86, 7.76) | 7.16 (6.79, 7.54) |
|  | Female | 8.50 (8.24, 8.76) | 8.66 (8.44, 8.89) |
|  | Non-binary | 10.79 (8.61, 12.96) | 9.28 (8.16, 10.39) |
|  | Prefer not to say | 10.63 (7.66, 13.60) | 7.62 (5.58, 9.66) |
| Well-being (SWEMWBS) | Male | 24.24 (23.87, 24.62) | 23.82 (23.51, 24.12) |
|  | Female | 23.29 (23.07, 23.51) | 22.76 (22.58, 22.94) |
|  | Non-binary | 23.62 (21.83, 25.41) | 22.17 (21.23, 23.10) |
|  | Prefer not to say | 21.31 (18.82, 23.79) | 23.02 (21.37, 24.67) |
| Sleep Quality (SCI-8) | Male | 20.39 (19.81, 20.98) | 21.16 (20.65, 21.66) |
|  | Female | 19.70 (19.37, 20.04) | 20.31 (20.01, 20.62) |
|  | Non-binary | 15.43 (12.65, 18.20) | 19.14 (17.68, 20.60) |
|  | Prefer not to say | 16.22 (12.33, 20.11) | 21.10 (18.37, 23.82) |

| **Supplementary Table 6**. Results from multivariable linear mixed effects models estimating the associations between taking the mental health literacy course and 12-week changes in student well-being and mental health by gender, age, and semester | | | | | | | | | | | | | |
| --- | --- | --- | --- | --- | --- | --- | --- | --- | --- | --- | --- | --- | --- |
|  | **Gender** | | | | | | | | | | | | p* |
|  | **Male** | | **Female** | | | | **Non-binary** | | | | **Prefer not to say** | |  |
| Anxiety Symptoms (GAD-7; 0-21) | -0.27 (-0.59, 0.04) | | **-0.45 (-0.61, -0.29)** | | | | 1.31 (-0.63, 3.24) | | | | -0.46 (-2.62, 1.69) | | 0.001 |
| Depressive Symptoms  (PHQ-9; 0-27) | -0.17 (-0.51, 0.17) | | **-0.39 (-0.56, -0.21)** | | | | 0.97 (-1.31, 3.25) | | | | **-2.78 (-5.06, -0.50)** | | <0.001 |
| Sleep Quality  (SCI-8; 0-32) | **0.56 (0.15, 0.97)** | | **0.78 (0.57, 0.98)** | | | | 1.50 (-1.17, 4.17) | | | | **5.39 (2.66, 8.11)** | | 0.001 |
| Well-being  (SWEMWBS; 7-35) | 0.20 (-0.08, 0.48) | | **0.24 (0.10, 0.38)** | | | | -1.22 (-2.93, 0.50) | | | | 1.66 (-0.27, 3.58) | | 0.009 |
|  | **Age Group** | | | | | | | | | | | |  |
|  | **≤17** | **18** | | **19** | | **20** | | **21** | | **22** | | **≥23** | p* |
| Well-being  (SWEMWBS; 7-35) | -0.15 (-0.72, 0.43) | **0.55 (0.31, 0.79)** | | 0.06 (-0.25, 0.37) | | 0.02 (-0.30, 0.33) | | 0.22 (-0.10, 0.55) | | **0.71 (0.13, 1.28)** | | 0.32 (-0.11, 0.75) | 0.038 |
|  | **Semester** | | | | | | | | | | | |  |
|  | **Fall 2021** | | | | **Fall 2022** | | | | **Fall 2023** | | | | p* |
| Anxiety Symptoms (GAD-7; 0-21) | -0.24 (-0.59, 0.11) | | | | **-0.49 (-0.83, -0.15)** | | | | **-1.02 (-1.39, -0.65)** | | | | 0.008 |
| Depressive Symptoms  (PHQ-9; 0-27) | -0.06 (-0.43, 0.31) | | | | 0.32 (-0.05, 0.68) | | | | -0.37 (-0.77, 0.02) | | | | 0.041 |
| Sleep Quality  (SCI-8; 0-32) | -0.05 (-0.58, 0.48) | | | | 0.22 (-0.21, 0.63) | | | | **1.04 (0.58, 1.50)** | | | | 0.004 |
| *Notes: (1) *p-value from Wald test for interaction terms, (2) Models adjusted for potential confounding, and interactions between time, gender, and course participation, (3) Only effects that were statistically significant are presented (multivariate Wald test p<.05), (4) Sample sizes (n) for course takers ranged from: Males (627-629), Female (2,181-2,182), Non-binary (29), Prefer not to say (13), Age ≤17 (95), Age 18 (583), Age 19 (490-492), Age 20 (501), Age 21 (498), Age 22 (140), Age ≥23 (542-543), Fall 2021 (302), Fall 2022 (353-354), Fall 2023 (516), (5) Sample sizes (n) for non-course takers ranged from: Males (939-1,071), Female (3,237-3,564), Non-binary (98-100), Prefer not to say (26-27), Age ≤17 (562-632), Age 18 (1,879-2,083), Age 19 (476-533), Age 20 (411-445), Age 21 (409-443), Age 22 (183-203), Age ≥23 (234-256), Fall 2021 (2,329-2,624), Fall 2022 (1,275-1,370), Fall 2023 (765-846).* | | | | | | | | | | | | | |
